# Surface Characteristic Effect of Ag/TiO_2_ Nanoarray Composite Structure on Supercapacitor Electrode Properties

**DOI:** 10.1155/2018/2464981

**Published:** 2018-07-24

**Authors:** Jie Cui, Lin Cao, Dahai Zeng, Xiaojian Wang, Wei Li, Zhidan Lin, Peng Zhang

**Affiliations:** ^1^Analytical and Testing Center of SCUT, South China University of Technology, Guangzhou 510640, China; ^2^Institute of Advanced Wear & Corrosion Resistant and Functional Materials, Jinan University, Guangzhou 510632, China

## Abstract

Ag-ion-modified titanium nanotube (Ag/TiO_2_-NT) arrays were designed and fabricated as the electrode material of supercapacitors for electrochemical energy storage. TiO_2_ nanotube (NT) arrays were prepared by electrochemical anodic oxidation and then treated by Ag metal vapor vacuum arc (MEVVA) implantation. The Ag amount was controlled via adjusting ion implantation parameters. The morphology, crystallinity, and electrochemistry properties of as-obtained Ag/TiO_2_-NT electrodes were distinguished based on various characterizations. Compared with different doses of Ag/TiO_2_-NTs, the electrode with the dose of 5.0 × 10^17^ ions·cm^−2^ exhibited much higher electrode capacity and greatly enhanced activity in comparison to the pure TiO_2_-NTs. The modified electrode showed a high capacitance of 9324.6 mF·cm^−3^ (86.9 mF·g, 1.2 mF·cm^−2^), energy density of 82.8 *μ*Wh·cm^−3^ (0.8 *μ*Wh·g, 0.0103 *μ*Wh·cm^−2^), and power density of 161.0 mW·cm^−3^ (150.4 *μ*W·g, 2.00 *μ*W·cm^−2^) at the current density of 0.05 mA. Therefore, Ag/TiO_2_-NTs could act as a feasible electrode material of supercapacitors.

## 1. Introduction

Nowadays, with the rapid development of science and technology, the depletion of fossil fuels urges a need for efficient, clean, and sustainable sources of energy, as well as with the demand for energy conversion and storage [1–3]. In recent years, supercapacitors have drawn great attention, mainly due to their high power and energy density and long lifecycle, which led to the high-power output and high-energy storage. The electrode material is one of the most important components that govern the overall electrochemical performance of the supercapacitor. Therefore, the development of high-performance electrode material is extremely important [4]. The critical problem is the energy density limitation, which has already hindered its extensive application. Hence, it is pressing to discover new electrode materials for enhancing high capacitance.

Aiming to improve the electrode material capacitance, the design of supercapacitor electrode materials starts with two aspects to solve the problem. One approach is to prepare a nanostructure with suitable pore-size distribution and pore network, which leads to high specific surface area, more active sites and high rates of ion diffusion, and a low internal electrical resistance for more efficiency at carrying electronic charges [5–7]. It would also bring a better electrochemical and mechanical stability for good cycling performance. It is crucial to match the size of solvated anions and cations by tuning the structure of the nanopores [8–10]. Another way is to design and prepare a composite material, which can combine the high-capacitance materials with large specific surface structure materials.

Among a large number of advanced supercapacitor electrode materials, metal oxides such as titanium oxides are considered as one of the most promising materials for the next generation of supercapacitors [11–14]. In general, metal oxides can provide higher energy density for supercapacitors than conventional carbon materials and better electrochemical stability than polymer materials [15]. They not only store the energy like electrostatic carbon materials but also exhibit the electrochemical faradaic reactions between electrode materials and ions within appropriate potential windows [16–18]. The metal oxides should be electronically conductive for the supercapacitor application [19–21]. For the electrochemical stability, the metal can exist in two or more oxidation states over a continuous range with no phase changes, which may involve structure changes to reduce the stability of electrodes [22–24]. The oxide lattice also can facilitate the protons intercalating freely into and out of the oxide during the reduction and oxidation processes, respectively [25].

TiO_2_-NTs with vertically oriented nanotube arrays can provide a direct pathway for electron transport along the nanotube's long axis to the substrate [26]. It provides a high surface area, which also shows excellent chemical stability. It also enhances the electrocatalytic activity, cycling stability, and charge rate performance of supercapacitors [27, 28]. Nevertheless, poor capacitive behaviour still limits the use of TiO_2_-NTs. Its large bandgap causes a poor efficiency and low conductivity. Therefore, it is necessary to improve the conductivity of TiO_2_. The electrical conductivity of TiO_2_ electrodes can be achieved in a composite electrode material by introducing a conductive substance into the oxide [29, 30].

Ag is considered as one of the prospective modified materials because of its relatively high conductivity. Herein, we report a composite supercapacitor electrode (Ag/TiO_2_-NT arrays) to improve the electric conductivity by introducing Ag into TiO_2_-NTs. Therefore, a modified large specific area and good conductive supercapacitor electrode can be obtained. The conventional anodization process was used to prepare TiO_2_-NTs, and the Ag was induced by ion implantation method. The morphologies, microstructures, and electrochemical performances of the Ag/TiO_2_-NT products were investigated.

## 2. Materials and Methods

### 2.1. Materials

All the chemicals used in this study were analytical reagent grade. Meanwhile, ammonium fluoride (NH_4_F) and ethylene glycol (EG) were obtained from Tianjin Damao Chemical Reagent Factory. Sodium sulfate (Na_2_SO_4_) and ethanol were provided by Guangzhou Chemical Reagent Factory. The pure titanium foil (99.6% pure), with a thickness of about 30 *μ*m, was purchased from Guangzhou Zhongyao Metal Technology Co. Ltd. Pure Ag (99.999% pure) was prepared for ion implantation target.

### 2.2. Fabrication of Ag/TiO_2_-NT Arrays

The TiO_2_-NT arrays were prepared by two-electrode electrochemical anodic oxidation. Before the anodization, the pure titanium foil was ultrasonically washed for 10 minutes in acetone, absolute ethanol, and deionized water. During the anodic oxidation process, the pretreated Ti foil was used as the anode and the stainless steel as the cathode, with an electrolyte which was composed of glycol (98 vol. %), deionized H_2_O (2 vol. %), and NH_4_F (0.3 wt. %). The oxidation voltage was 40 V, and the anodic oxidation time was 1 h. After that, the anodized TiO_2_-NT arrays were treated with Ag-ion implantation with an accelerating voltage of 60 kV and the different ion implantation dose. Then, Ag/TiO_2_-NT samples were obtained by adjusting the implanting doses of 0.5, 1.0, 5.0, and 10.0 × 10^17^ ions·cm^−2^.

### 2.3. Characterization

Morphological observations were carried on a field-emission scanning electron microscope (FE-SEM, Zeiss, Oberkochen, Germany) and a field-emission transmission electron microscope (TEM, JEOL 2100F, Japan). Electrochemical measurements, such as galvanostatic charge–discharge (GCD), cyclic voltammetry (CV), and electrochemical impedance spectroscopy (EIS, 100 kHz to 0.01 Hz), were tested by an electrochemical workstation (Princeton PARSTAT 4000). The electrochemical performance of individual electrodes was tested by a three-electrode system with a 0.5 M Na_2_SO_4_ solution serving as an electrolyte; a platinum electrode and a saturated calomel electrode (SCE) were used as a counter electrode and reference electrode, respectively.

## 3. Results and Discussion

### 3.1. Effect of Ag-Ion Implantation on the Microstructure of TiO_2_-NTs

**Figure 1:**
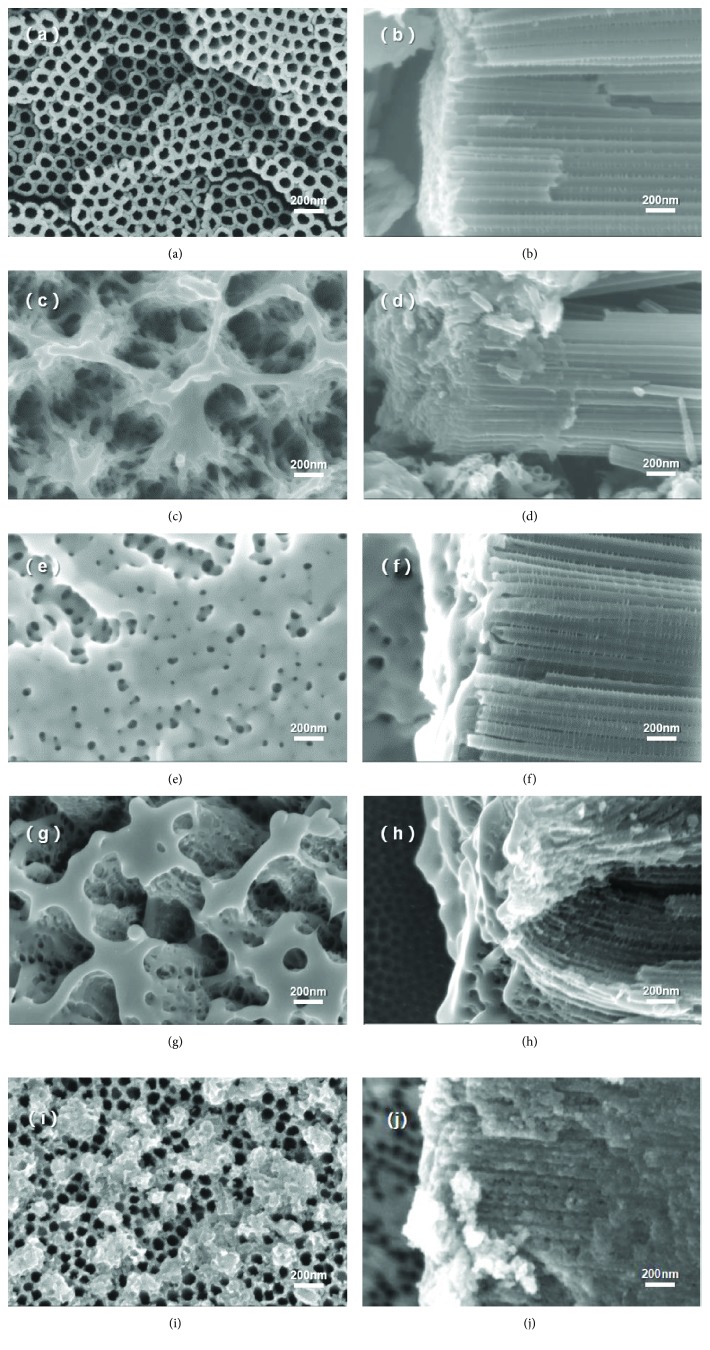
SEM images of Ag/TiO_2_ electrode at different doses of Ag-ion implantation: top-view of (a) 0 ions·cm^−2^, (c) 0.5 × 10^17^ ions·cm^−2^, (e) 1.0 × 10^17^ ions·cm^−2^, (g) 5.0 × 10^17^ ions·cm^−2^, and (i) 10.0 × 10^17^ ions·cm^−2^; side-view of (b) 0 ions·cm^−2^, (d) 0.5 × 10^17^ ions·cm^−2^, (f) 1.0 × 10^17^ ions·cm^−2^, (h) 5.0 × 10^17^ ions·cm^−2^, and (j) 10.0 × 10^17^ ions·cm^−2^.

Modified surface morphologies of Ti fossil after anodic oxidation and ion implantation with the Ag dose of 0 ions·cm^−2^ (pure TiO_2_-NTs), 0.5 × 10^17^ ions·cm^−2^, 1.0 × 10^17^ ions·cm^−2^, 5.0 × 10^17^ ions·cm^−2^, and 10.0 × 10^17^ ions·cm^−2^ are shown in Figure 1. As shown in Figures 1(a) and 1(b), the surface morphology of TiO_2_-NTs presented as a nanotube array structure. And the average inner diameter of the tube is about 50 nm. After Ag-ion implantation, the surface morphology changed a lot. A net-like surface layer with uniform pores was formed after the Ag-ion implantation dose of 0.5 × 10^17^ ions·cm^−2^ (as Figures 1(c) and 1(d) showed). The outermost layers of the NT wall were destroyed, and partial NT disconnected and reconnected into a network-like structure. The inside wall of the tube became a little rougher, which may further increase the surface-specific surface area. Although the top surface was changed a lot, it can be seen that the nanotube structure in the whole length is similar with Figure 1(b) as shown in the section view in Figure 1(d). Quite different with the surface of TiO_2_-NT arrays, the pipe orifices were blocked as shown in Figures 1(e) and 1(f), which was caused by the partial TiO_2_-NTs connected during the heat effect in ion implantation while the Ag-ion implantation dose is up to 1 × 10^17^ ions·cm^−2^. Besides that, a large number of much finer pores were generated. It can also be found that the length of NTs was reduced obviously as shown in the section view (Figure 1(f)) due to the surface TiO_2_ wall being involved in the generation of the new surface. The top nanotubes' lamellar “gap-” like structure is formed by the heat effect increased with the ion bombardment. And the bottom secondary nanotube grew under the action of high temperature. Therefore, the nanotube narrowed into a closed aperture. The surface was fully covered by a nanoisland-like structure as shown in Figure 1(g), when the Ag dose was up to 5.0 × 10^17^ ions·cm^−2^. Meanwhile, many nanopores were generated on the wall. Hence, the wall of NTs became much rougher as shown in Figure 1(h). When the Ag-ion implantation dose reached 10.0 × 10^17^ ions·cm^−2^ (Figures 1(i) and 1(j)), some globular particles appeared and distributed near the top of the nanotubes. It is possible that a part of the nanotube was destroyed; a lot of porous debris formed under the large number of ion bombardment. The cross-section observation showed that the wall of NTs became much rougher than that of the lower Ag dose. And the wall near the surface almost collapsed under such a high Ag dose. According to the surface and section analysis, it can be inferred that there were two opposite processes, a formation growth process by heat effect and a damage process by high-energy ion bombardment. Therefore, the island-like structure was formed on the surface occurring with the surface layer growth and destruction. At the same time, the nanopores have been created by ion bombardment damage under high Ag dose. And it may lead to higher specific surface area. Meanwhile, the length of the nanotube was further shortened while the Ag dose increased as shown in Figure S3 in the supplementary material. It can be inferred that there is a saturated dose by ion implantation method. When the implantation was lower than the saturated dose, ions were just injected into the surface. Meanwhile, a new phase or new structure was generated by the heat effect and the joining of the new ion. Once the implantation dose was oversaturated, the matrix structure may be damaged due to the overheat effect and excessive ion bombardment.

**Figure 2:**
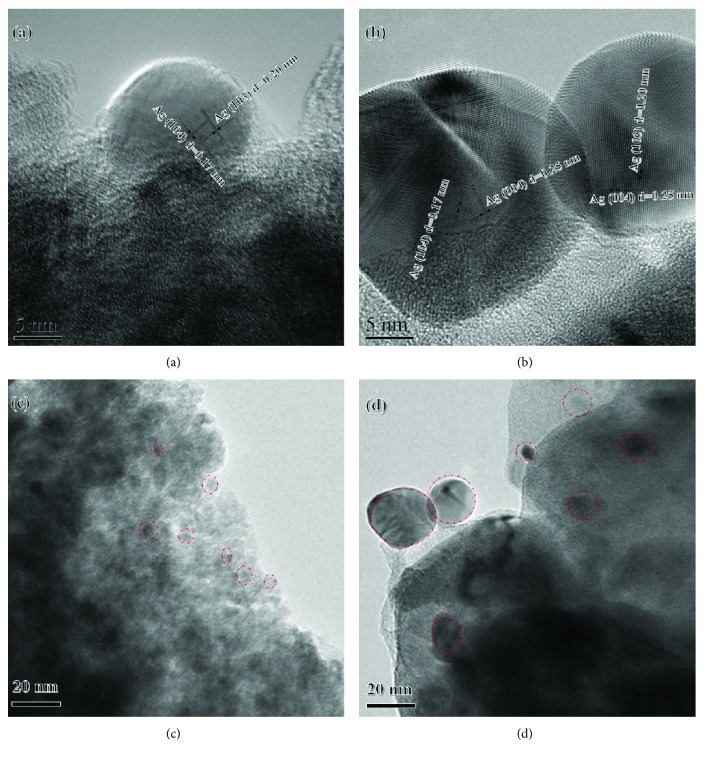
HRTEM and TEM bright images of the Ag/TiO_2_ electrode at different doses of Ag-ion implantation: (a, c) 5.0 × 10^17^ ions·cm^−2^; (b, d) 10.0 × 10^17^ ions·cm^−2^.

In order to further investigate the new phase after Ag-ion implantation, the microstructure of the Ag-ion implantation dose under 5.0 × 10^17^ ions·cm^−2^ and 10.0 × 10^17^ ions·cm^−2^ was also observed by transmission electron microscopy (TEM), high-resolution TEM (HRTEM), high-angle annular dark field (HAADF), and energy-dispersive X-ray detector (EDX). It can be proved that Ag nanoparticles were created both in this two samples as shown in Figures 2(a) and 2(b). It can also be found that the spherical particle as marked in red dotted circular areas is found both in these two samples as shown in Figures 2(c) and 2(d), which is consistent with the high-magnification SEM images as shown in Figure S1 in the support information (SI). And the particle sizes in these two samples are 10–15 nm and 20–35 nm, respectively, which supports that particle size of Ag is increased while the implantation dose is up to 10.0 × 10^17^ ions·cm^−2^. Besides that, it can also be found that the matrix of TiO_2_-NTs shows partial amorphous as shown in Figure 2(b) while the dose is up to 10.0 × 10^17^ ions·cm^−2^. This is very different with the crystalline TiO_2_ in the sample under the dose of 5.0 × 10^17^ ions·cm^−2^. Besides that, the EDX of these two samples as shown in Figure 2 in SI shows that the Ag is homogeneously distributed in the TiO_2_-NT matrix.

**Figure 3:**
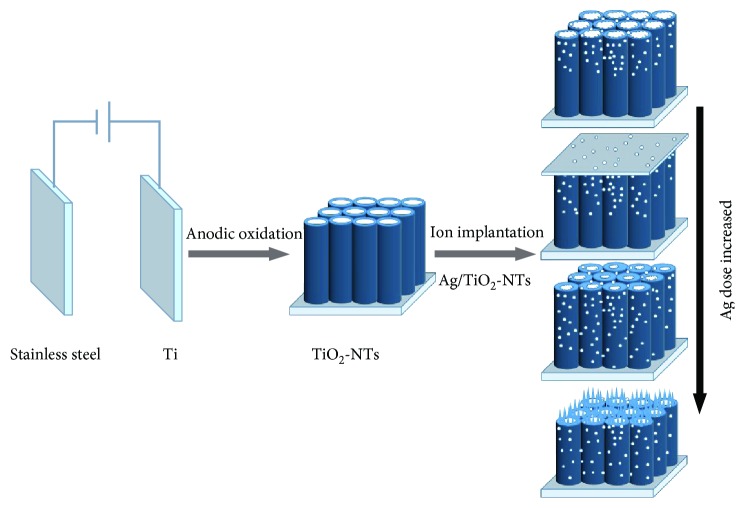
Schematic illustration for the synthesis process of the Ag-ion implantation on TiO_2_-NTs.

Hence, it can be briefly summarized that there is a relationship between the structure and Ag implantation as shown in Figure 3. Firstly, TiO_2_-NT array structures were formed on the Ti foil matrix after electrochemical anodization in a two-electrode system. Then a few nano-Ag particles were generated, and the surface was modified under lower Ag dose. While the Ag dose increased continuously, more Ag particles formed on the TiO_2_-NT arrays and the length of the tube was shortened. However, the TiO_2_-NT array matrix was damaged, and the particle size of Ag increased under the oversaturated Ag dose.

### 3.2. Effect of Ag/TiO_2_ Electrode Conductivity by Ion Implantation

**Figure 4:**
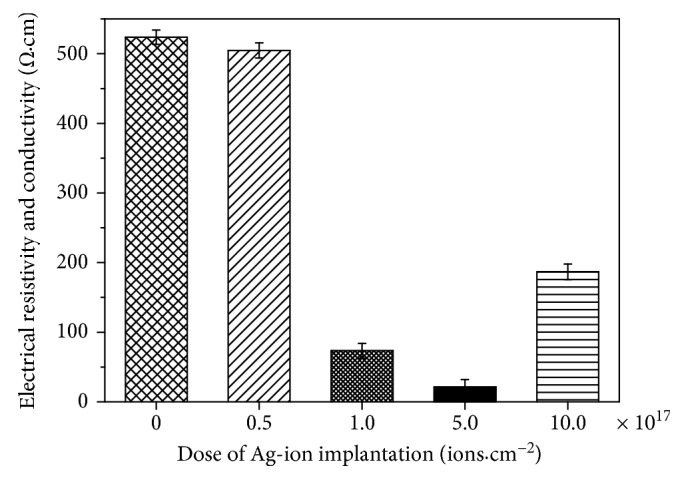
The resistivity of Ag/TiO_2_ electrodes at different doses of Ag-ion implantation.

In order to research the conductive properties of Ag/TiO_2_ electrodes, the four-point probe resistance meter method was chosen. Electrode resistivity with different Ag implantation doses was obtained as shown in Figure 4. Compared with the unmodified samples, the resistivity of the sample was decreased significantly with the increase of ion implantation dose. It can be found that resistivity of the Ag/TiO_2_ electrode reduced from 523.7 Ω·cm (the unmodified NTs) to 21.6 Ω·cm; the minimum resistivity sample had a corresponding Ag dose of 5.0 × 10^17^ ions·cm^−2^. Hence, it is no doubt that Ag-ion implantation modification can greatly improve the conductivity especially under the dose of 5.0 × 10^17^ ions·cm^−2^. In addition, although the resistivity increased a bit more when the dose increased to 10.0 × 10^17^ ions·cm^−2^, it is still much smaller than that of unmodified samples. Obviously, the slight increased resistivity may be impacted by the almost blocked and damaged tubes under the oversaturated dose. Therefore, the appropriate Ag dose with smaller particle size and integrated array structure may contribute to enhance the conductivity and smoothen the passage of the electronic transmission channel, which can finally improve the conductivity of the Ag/TiO_2_-NT electrodes.

### 3.3. Effect on Ag/TiO_2_ Electrode Electrochemical Properties by Ion Implantation

**Figure 5:**
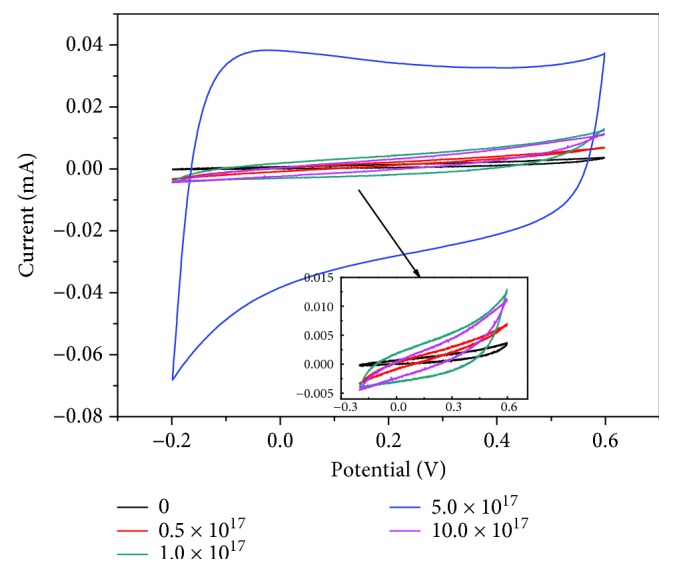
CV curves of Ag/TiO_2_ electrodes at different doses of Ag-ion implantation (100 mV·s^−1^).

**Figure 6:**
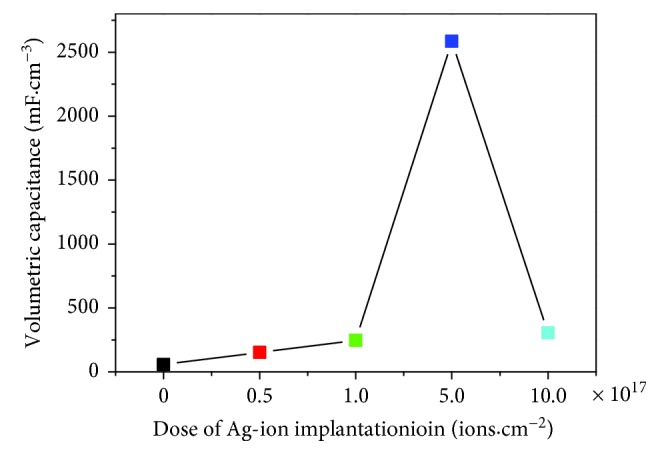
Relationship between the dose of Ag-ion implantation and specific capacitance.

The CV curves of the Ag/TiO_2_ composite electrode with different Ag ions were obtained at the sweep speed of 100 mV·s^−1^ as shown in Figure 5. The CV curve shape of the Ag/TiO_2_ electrode is rectangular, which stands for a good capacitor performance. That is because the improved electrical conductivity is beneficial a lot to the electrochemical reaction in the process of rapid transmission and transfer of electric charge. The significantly increased integral area of the CV curves illustrates the significantly enhanced electrode electrochemical activity and the effectively improved capacitance. The specific capacitance was calculated as shown in Figure 6 by CV curves under different Ag-ion implantation dosages. The electrochemical activity of the Ag/TiO_2_ electrode changed with the increase of the Ag-ion implantation dose. And the variation trend is consistent with the change of electrode conductivity, which is also increased with the Ag dose lower than 5.0 × 10^17^ ions·cm^−2^ and then decreased with the reincreased Ag dose. Therefore, the Ag/TiO_2_ electrode with a dose of 5.0 × 10^17^ ions·cm^−2^ shows the best electrochemical activity while the resistance rate is the minimum, which is contributed by the appropriate Ag doping with smaller particle size and smooth passage of the electronic transmission channel. It can also be found obviously that the area of the CV curve and the capacitance is decreased when the dose of Ag ions is 10.0 × 10^17^ ions·cm^−2^ which is also consistent with the change of resistivity. This indicates again that the Ag dose of 5.0 × 10^17^ ions·cm^−2^ is the most suitable dose, which leads to an appropriate Ag doping and a relatively complete NT array structure. In short, the above experimental results show that Ag-ion implantation is a very effective measure to improve the conductivity and electrochemical activity of Ag/TiO_2_ electrodes. And the most appropriate Ag dose is 5.0 × 10^17^ ions·cm^−2^ in the presented study.

**Figure 7:**
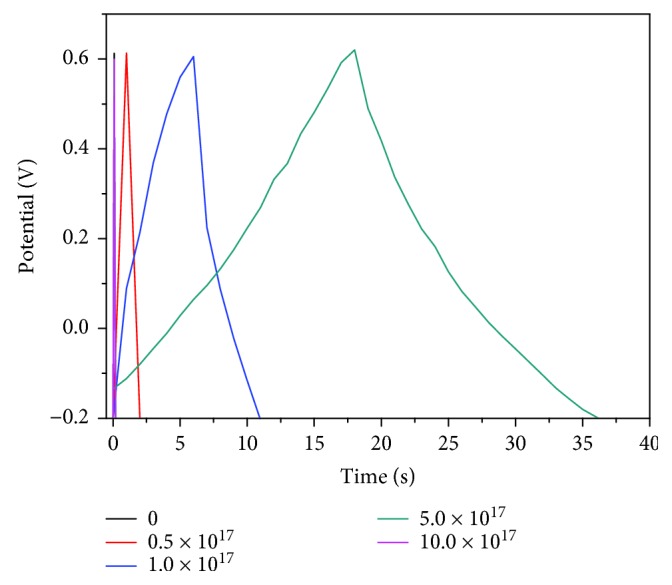
GCD curve of Ag/TiO_2_ electrodes at different doses of Ag-ion implantation.

**Figure 8:**
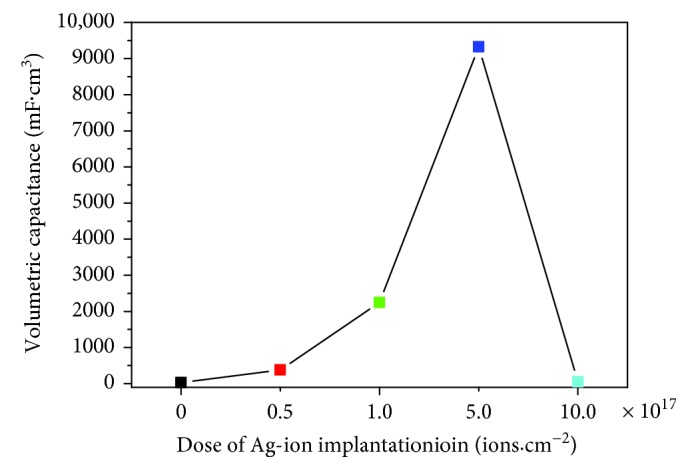
Relationship between different doses of Ag-ion implantation and specific capacitance.

The charge–discharge curve (GCD curve) in Figure 7 also shows that the electrochemical activity of the electrode is enhanced with the improvement of electrode conductivity, and the charge and discharge performance is also improved significantly. The Ag/TiO_2_ electrode GCD curve shows a good triangular shape (Figure 7) under different Ag-ion implantation dosages, when the current density is 0.05 mA·cm^−2^. The curve also has a good linear relationship between potential and time. It reveals that the electrode materials have a good coulombic efficiency. With the increase of the Ag-ion implantation dose, the electrode material needs a longer discharge time, which indicates that the electrode material shows a larger capacity to store energy. The ratio of capacitance can be calculated by the GCD curve as shown in Figure 8. The electrode without Ag-ion implantation is only 32.9 mF·cm^−3^ (0.00625 mF·cm^−2^), while the modified ones are 378.8, 2246.7, 9324.6, and 54.3 mF·cm^−3^ (0.0625, 0.344, 1.156, and 0.00625 mF·cm^−2^, resp.), with the corresponding dose of 0.5 × 10^17^ ions·cm^−2^, 1.0 × 10^17^ ions·cm^−2^, 5.0 × 10^17^ ions·cm^−2^, and 10.0 × 10^17^ ions·cm^−2^. The specific capacitance of the 5.0 × 10^17^ ions·cm^−2^ sample is increased about 282.4% much more than the untreated one.

**Figure 9:**
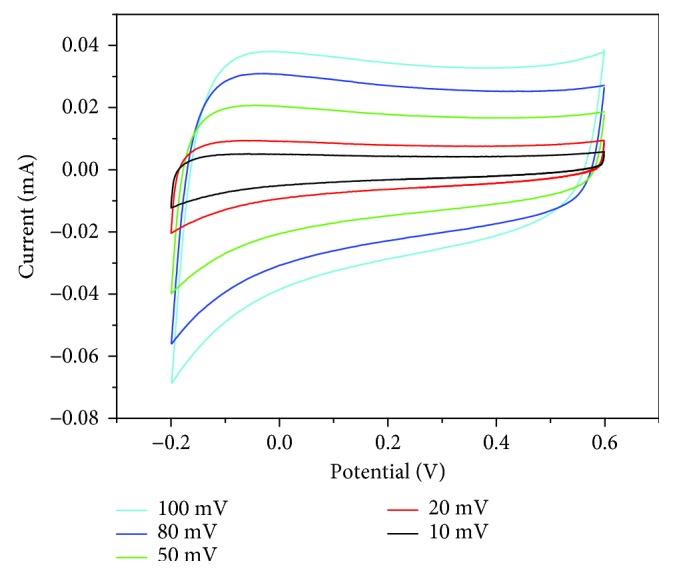
CV curves with different scan rates for Ag-ion implantation dose of 5.0 × 10^17^ ions·cm^−2^.

**Figure 10:**
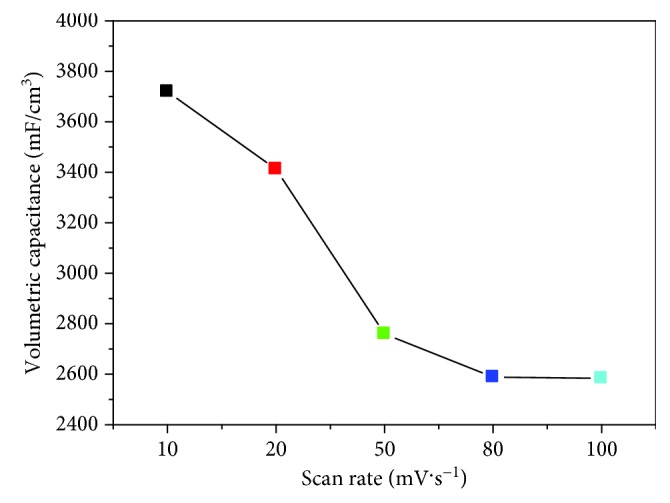
Relationship between different scan rates and specific capacitance for Ag-ion implantation dose of 5.0 × 10^17^ ions·cm^−2^.

In order to further verify the performance of the Ag/TiO_2_ electrodes with the dose of 5.0 × 10^17^ ions·cm^−2^, the CV test was performed at different scanning rates (Figure 9). During the −0.2~0.6 V voltage range, the shape of the CV curve basically keeps consistent with the increase of the scanning speed. The Ag/TiO_2_ electrode CV curves still keep a good rectangular shape with a larger integral area at a high scan speed (100 mV·s^−1^). The large specific capacitance shows that the electrode electrochemical performance is improved obviously after modified ion implantation. With the scanning speed increased, the specific capacitance of electrode materials is also decreased. But the specific capacitance remains at a rate of 69.5% (Figure 10), while the sweep speed from 10 up to 100 mV·s^−1^. It shows that the electrode response performance to voltage variation is relatively good, and the electrode owns a good ratio performance.

**Figure 11:**
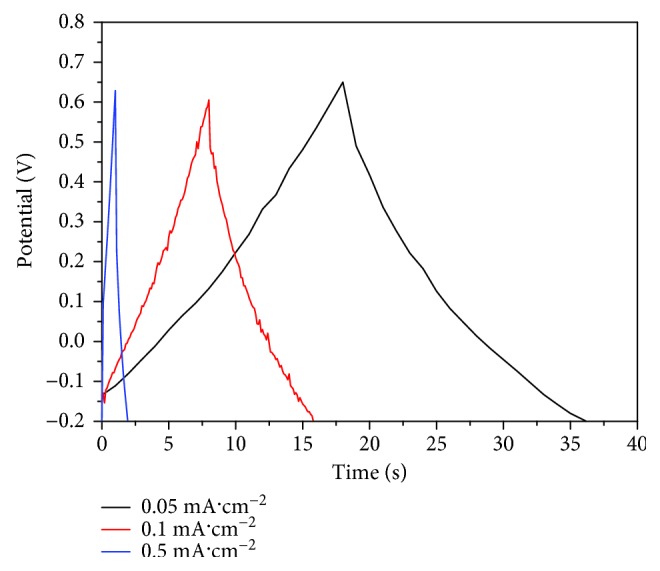
GCD curves with different current densities for Ag-ion implantation dose of 5.0 × 10^17^ ions·cm^−2^.

**Figure 12:**
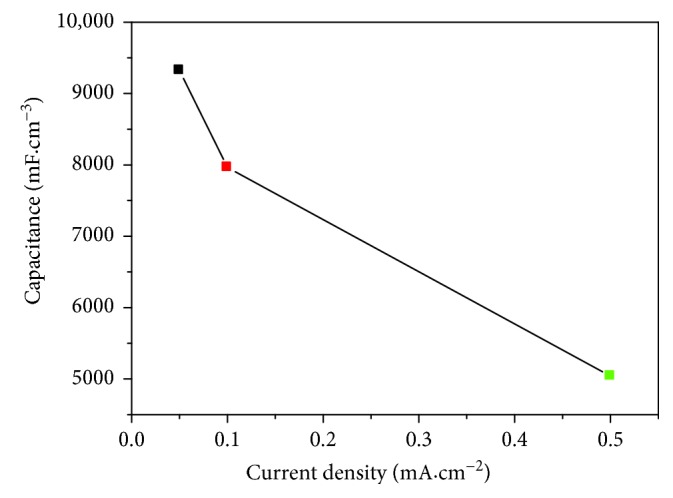
Area-based magnification curve for Ag-ion implantation dose of 5.0 × 10^17^ ions·cm^−2^.

The GCD curves of the Ag/TiO_2_ electrode with an Ag dose of 5.0 × 10^17^ ions·cm^−2^ at different current densities are shown in Figure 11. The electrode charge and discharge curves of the electrode show a good symmetry and linear properties under different current densities, which means that the electrode has a high coulombic efficiency. When the electrode under the given current density at 0.05, 0.1, and 0.5 mA·cm^−2^, the specific capacitance is 9324.6, 7963.7, and 5040.3 mF·cm^−3^ (1.156, 0.988, and 0.625 mF·cm^−2^) (Figure 12), respectively, which remains at a rate of 54.0%. After the Ag-ion implantation is modified, the conductivity of the electrode was improved. And the electron can be quickly transmitted to the TiO_2_-NTs at a large current charge and discharge process, in which ratio performance has no sharp attenuation. The modified electrode owns a good capacitance characteristic.

**Figure 13:**
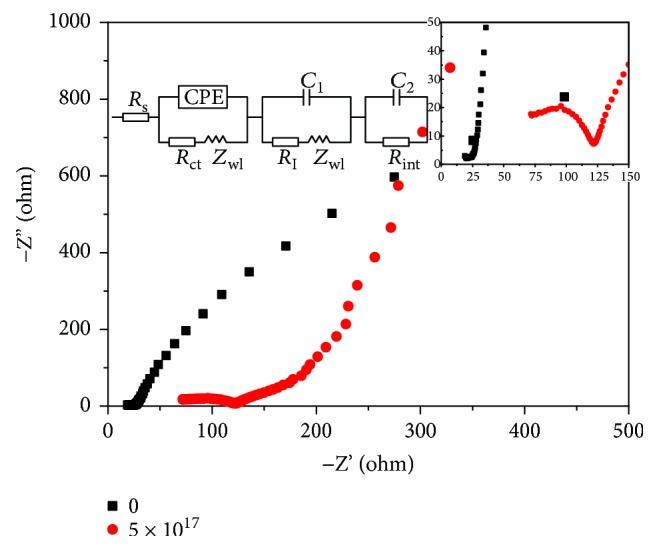
Impedance curve of Ag-ion implantation dose of 5.0 × 10^17^ ions·cm^−2^.

An electrochemical impedance test was used to evaluate the conductivity and ion transfer capacity of the supercapacitor electrode material. The Ag/TiO_2_ electrode impedance diagram with the Ag dose of 5.0 × 10^17^ ions·cm^−2^ is shown in Figure 13. The semicircle diameter of the high-frequency zone is small, which indicates that the internal resistance of the electrode material is very small after the modification of Ag-ion implantation. The straight line is close to 90° of the low-frequency area, which shows a better electrode capacitance performance. Nyquist plots in Figure 13 illustrate the impedance characteristics of the TiO_2_/Ti and Ag/TiO_2_/Ti electrodes in 0.5 M Na_2_SO_4_ solution. According to the Randles circuit in Figure 13, the intercept on the real axis represents the series resistance (*R*_s_); this impedance is contributed from the contact resistance, bulky electrolyte, and electrode. The arc arises from the charge-transfer resistance (*R*_ct_) at the electrode/electrolyte interface, whereas *Z*_w_ represents the Nernst diffusion impedance corresponding to the diffusion resistance of the redox species. CPE is a constant phase element, and it is frequently used as a substitute for capacitors in an equivalent circuit to fit the impedance behaviour of the electrical double layer. The *R*_ct_ values of the TiO_2_/Ti and Ag/TiO_2_/Ti electrodes are 310,000 and 74.79 Ω·cm^2^, respectively, whose values can be employed to assess the charge-transfer ability of the electrodes. A lower *R*_ct_ corresponds to facile charge-transfer kinetics within electrodes; therefore, the Ag/TiO_2_/Ti electrode has the highest charge-transfer capacity because of its high electrical conductivity.

## 4. Conclusions

In summary, an Ag-ion implantation modification Ag/TiO_2_-NT array composite structure has been successfully synthesized via two steps: anodization and ion implantation. The surface morphology, conductivity, electrochemical properties under different Ag implantation dose was studied. An Ag/TiO_2_ electrode was prepared, and we got the following conclusions:

1. The surface morphology of TiO_2_-NTs had great changes by Ag-ion-implantation-modifying method. With different Ag-ion implantation doses, the surface morphology of Ag/TiO_2_ electrodes was affected by the Ag-ion implantation process heating effect on the growth of the surface film layer and the high-energy ion bombardment of surface-coating damage
2. Ag-ion implantation has great benefit to the improvement of the Ag/TiO_2_ electrode conductivity. When the Ag-ion implantation dose was 5.0 × 10^17^ ions·cm^−2^, a minimum resistivity (21.6 Ω·cm) was obtained, which had fallen by about 95.8% compared with the sample without ion implantation modification.
3. Ag ions were implanted into TiO_2_ nanotube array structures; they can enhance the conductivity and smoothen the passage of the electronic transmission channel. When the Ag/TiO_2_ electrode was charged at 0.05 mA·cm^−2^, and the implantation dose of Ag ion was 5.0 × 10^17^ ions·cm^−2^, the specific capacity of the electrode could reach up to 9324.6 mF·cm^−3^ (1.156 mF·cm^−2^). The ratio property can reach 54.0% with the current density increased from 0.05 to 0.5 mA·cm^−2^.

Because of the strong synergistic effect between the Ag-NPs and the TiO_2_ nanotube array structure in this study, this high-performance Ag/TiO_2_ nanotube array structure electrode material is expected to have potential applications in electrochemical energy storage devices.

## Data Availability

All data generated or analysed during this study are included in this article and its supplementary information files.

## Supplementary Materials

Supplementary Materials Figure S1: nano-Ag-NPs on the surface of the Ag/TiO_2_ electrode with different doses of Ag-ion implantation: (a) 5 × 10^17^ ions·cm^−2^; (b) 1 × 10^18^ ions·cm^−2^. Figure S2: elemental mapping by EFTEM analysis for Ag/TiO_2_ electrode with different doses of Ag-ion implantation: (a, b) 5 × 10^17^ ions·cm^−2^; (c, d) 1 × 10^18^ ions·cm^−2^. Figure S3: section view and nanotube length of Ag/TiO_2_ electrode with different doses of Ag-ion implantation: (a) 0 ions·cm^−2^; (b) 5 × 10^16^ ions·cm^−2^; (c) 1 × 10^17^ ions·cm^−2^; (d) 5 × 10^17^ ions·cm^−2^; (e) 1 × 10^18^ ions·cm^−2^.
