# Supplementary material for: Surface Characteristic Effect of Ag/TiO2 Nanoarray Composite Structure on Supercapacitor Electrode Properties
Source: Scanning. 2018 Jul 24;2018:2464981. doi: 10.1155/2018/2464981 (PMC6081553; doi:10.1155/2018/2464981)
Supplement: Supplementary Materials — Figure S1: nano-Ag-NPs on the surface of the Ag/TiO2 electrode with different doses of Ag-ion implantation: (a) 5 × 10^17^ ions·cm−2; (b) 1 × 10^18^ ions·cm−2. Figure S2: elemental mapping by EFTEM analysis for Ag/TiO2 electrode with different doses of Ag-ion implantation: (a, b) 5 × 10^17^ ions·cm−2; (c, d) 1 × 10^18^ ions·cm−2. Figure S3: section view and nanotube length of Ag/TiO2 electrode with different doses of Ag-ion implantation: (a) 0 ions·cm−2; (b) 5 × 10^16^ ions·cm−2; (c) 1 × 10^17^ ions·cm−2; (d) 5 × 10^17^ ions·cm−2; (e) 1 × 10^18^ ions·cm−2. [file 2464981.f1.doc]

# Scanning

**Surface characteristics effect of Ag/TiO_2_ nanoarray composite structure on supercapacitors electrode properties**

**Jie Cui^1^, Lin Cao^2^, Dahai Zeng^2^, Xiaojian Wang^2^, Wei Li^2^, Zhidan Lin^2,*^, Peng Zhang^2,*^**

^1^ Analytical and Testing Center of SCUT, South China University of Technology, Guangzhou, 510640, China.
^2^ Institute of Advanced Wear & Corrosion Resistant and Functional Materials, Jinan University, Guangzhou, 510632, China.

^*^Correspondence should be addressed to Zhidan Lin and Peng Zhang; [linzd@jnu.edu.cn](mailto:linzd@jnu.edu.cn); [tzhangpeng@jnu.edu.cn](mailto:tzhangpeng@jnu.edu.cn)

## Supplementary Materials

In order to understand the morphology of Ag after ion implantation, we made a magnified SEM image of the surface of Ag/TiO_2_ electrode with different doses of Ag-ion implantation as shown in figure S1. Nano Ag particles (Ag-NPs) were found on the surface, some were embedded in the surface, while some were implanted into the wall of nano tube. While the implantation dose upto 1×10^18^ ions·cm^-2^, the size of Ag-NPs was bigger than the 5×10^17^ ions·cm^-2^ one.


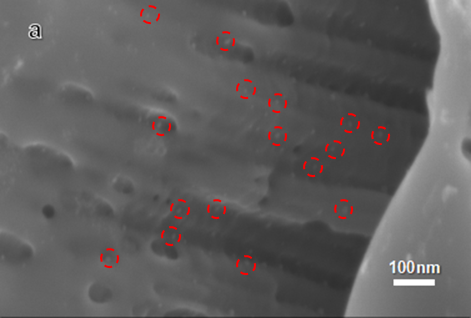

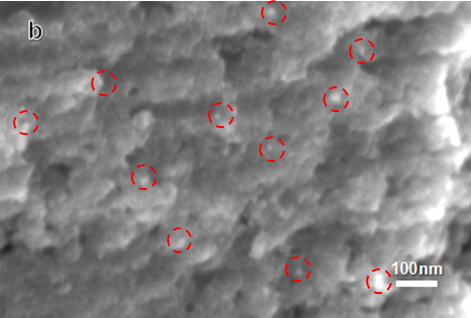


Figure S1 Nano Ag-NPs on the surface of Ag/TiO_2_ electrode with different doses of Ag-ion implantation, (a) 5×10^17^ ions·cm^-2^; (b) 1×10^18^ ions·cm^-2^;

These nano particles were verified by EFTEM analysis for Ag/TiO_2_ electrode with different doses of Ag-ion implantation as shown in figure S2. Elemental mapping results showed these nano particles were really Ag.


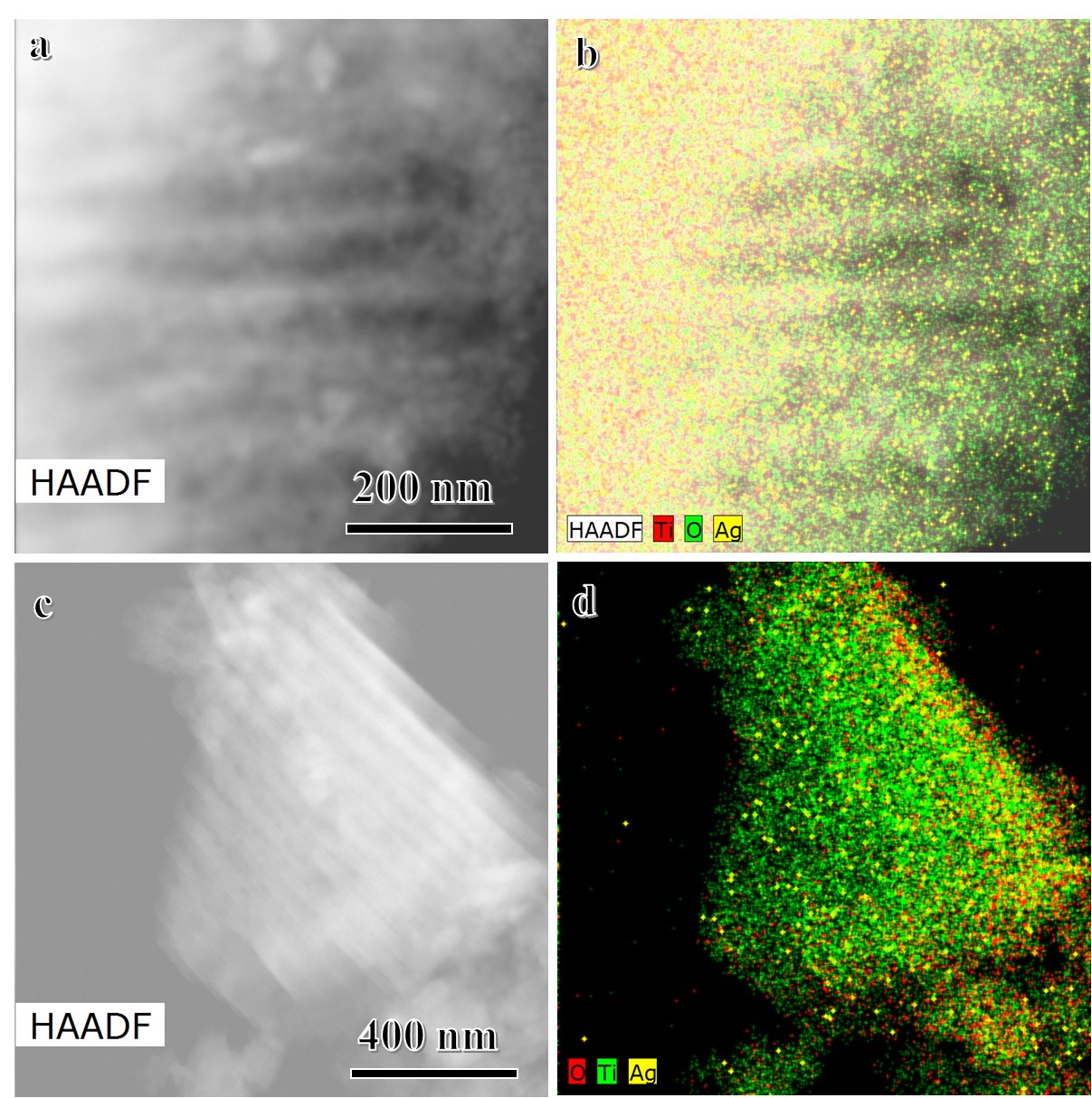


Figure S2 Elemental mapping by EFTEM analysis for Ag/TiO_2_ electrode with different doses of Ag-ion implantation, (a, b) 5×10^17^ ions·cm^-2^; (c, d) 1×10^18^ ions·cm^-2^;

The capacitance of Ag/TiO_2_ electrode was calculated by volume, the lengths with different doses of Ag-ion implantation were shown in figure S3.


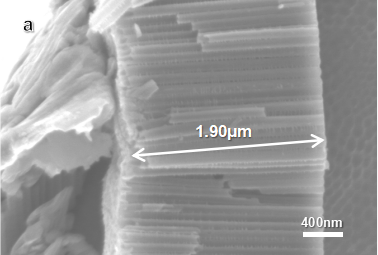

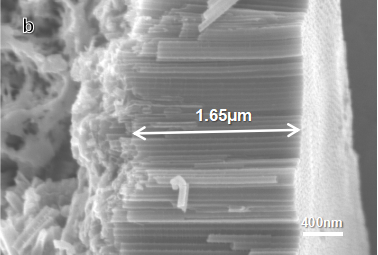


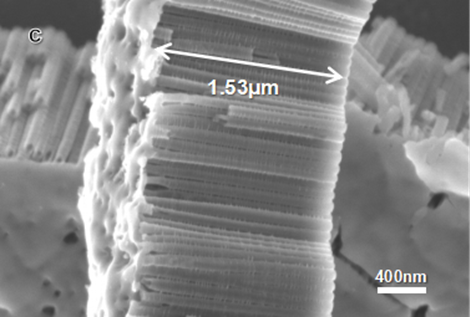

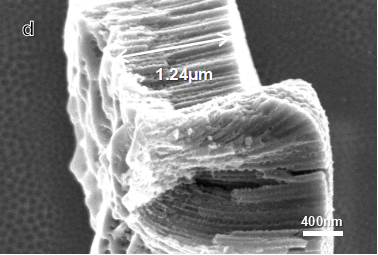


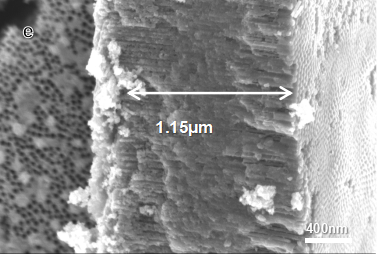


Figure S3 Section view and nanotube length of Ag/TiO_2_ electrode with different doses of Ag-ion implantation, (a) 0 ions·cm^-2^; (b) 5×10^16^ ions·cm^-2^; (c) 1×10^17^ ions·cm^-2^; (d) 5×10^17^ ions·cm^-2^; (e) 1×10^18^ ions·cm^-2^;
